# HPV-genotyping versus conventional cervical cytology as a screening method to detect dysplastic cervical epithelial changes

**DOI:** 10.1038/s41598-022-22438-z

**Published:** 2022-10-24

**Authors:** Mahmoud Abbas, Jan de Jonge, Olaf Bettendorf

**Affiliations:** 1 Gerhard-Domagk Institute for Pathology-University Muenster-Germany, Albert-Schweitzer-Campus 1-D17, 48149 Muenster, Germany; 2 Institute for Pathology and Cytology (IPN)-Schuettorf-Germany, Technikerstrasse 14, 48465 Schuettorf, Germany

**Keywords:** Cervical cancer, Health care, Oncology

## Abstract

The world health organization (WHO) called for coordinated global action in 2018 to eliminate cervical cancer, ensuring that every woman is screened and treated for precancerous lesions (World Health Organization. Cervical cancer: an NCD we can overcome. Geneva, 2018. http://www.who.int/director-general/speeches/detail/cervical-cancer-an-ncd-we-can-overcome.tegy). Cytology-based screening has been for decades the conventional method of screening. Ancillary techniques have been added like immunocytochemistry with P16/Ki67 and L1-Capsid, but these methods require maintenance of complex infrastructure and highly trained personnel as well as relatively short screening intervals. HPV-based screening method to detect high-risk groups is a faster and automated method, which does not need morphologically highly qualified personal with high social costs. In the study, we have focused on the distribution of cervical lesions in the age groups with concordance of detection HPV high-risk subtypes (HPV-HR) and on the safety of the screening method. In the Institute for Pathology and Cytology-Schuettorf-Leer-Germany 146.800 samples of women from the age of and above 35 years were analyzed between the beginnings of 2020 until the beginning of 2021. 63.710 cases under 35 years old were analyzed. The samples were processed for both conventional cytological techniques and for molecular detection and subtyping of HPV-HR according to the advice and measurements of BD-manufacture. In this study, we have studied the histopathological results (Table 2) after colposcopy according to the age subgroups. The histopathological results were subdivided into no dysplasia, cervical intraepithelial neoplasia I (CIN I), cervical intraepithelial neoplasia II (CIN II), cervical intraepithelial neoplasia III (CIN III), squamous cell carcinoma (Sq.c.c), adenocarcinoma in situ (AIS), endometrial carcinoma, endocervical adenocarcinoma and cases without biopsy during the colposcopy (COB). We have used the muenchener classification III (Table 3) as a subgrading system for the cytological specimens. The frequency of detecting HPV56/59/66 is higher as detecting HPV-16 and HPV18 in age groups under 35 years old, 41–50 years old and 51–60 years old. HPV16 is detected higher in age groups 35–40 years old and above 60 years. The incidence of high squamous intraepithelial lesions (CIN II and III) is 0.92% in age group 35–40 years, 0.54% in age under 35 years, 0.59% in age group 41–50 years old, 0.35% in age group 51–60 years old and 0.15% in age group above 60 years old. There is no significance (*p* value = 0.4060). Low grade cervical lesions (CIN I) were 0.13% (< 35 Ys), 0.35% (35–40 Ys), 0.36% (41–50 Ys), 0.25% (51–60 Ys) and 0.098% (> 60Y s), which was statistically significant (*p* value = 0.04,0.60). Without dysplasia 0.19% (< 35 Ys), 0.5% (35–40 Ys), 0.56% (41–50 Ys), 0.51 (51–60 Ys) and 0.26% (> 60 Ys). There is no significance between occurrence of cervical dysplasia and without dysplasia despite of detection of HPV-HR subtypes (*p* value = 0.1754). The only use of HPV-subtyping is not a secure method and a protective way for women. There are worldwide many HPV-positive cases, which have been psychologically impaired with higher costs, although they have no cervical epithelial changes during the HPV-infection. There are many HPV-negative cases, in some studies up to 13% of cases, which develop cervical cancer. We have the opinion and are convinced that the screening should be both morphologically via cytological examination and may be with adding immunocytochemistry to detect the really dysplastic cervical lesions. HPV-subtyping may be added every three years to detect the concomitant subtype.

## Introduction

The world health organization (WHO) called for coordinated global action in 2018 to eliminate cervical cancer, ensuring that every woman is screened and treated for precancerous lesions^1^. Cytology-based screening has been for decades the conventional method of screening. Ancillary techniques have been added like immunocytochemistry with P16/Ki67 and L1-Capsid, but these methods require maintenance of complex infrastructure and highly trained personnel as well as relatively short screening intervals. Immunocytochemistry has raised the sensitivity and specificity of the diagnosis but is still in many countries not well established^2^. The molecular detection of HPV high risk groups in women above 30–35 years has been proposed as a method of accuracy with higher sensitivity to detect women with risk to develop precancerous lesions. The advantage of this method is that it is a machinery automated work without the need to have highly trained and highly qualified personnel, but it is expensive. Due to lower specificity, HPV-based screening should not be done under the age of 35 years old^3^. In Germany at the beginning of 2020, a co-test-based screening program was applied for women at and above 35 years, which means performing conventional cytological analysis and HPV-HR-test for each woman above 35 years and in some cases under 35 years old. This is a retrospective study and experience after one year with the new system in Germany. This study will analyze, which of the methods give both clinicians and patients security to avoid progression of cervical dysplasia to cervical carcinoma for an early detection of cell changes under human papilloma infection. It will analyze, which HPV-subtypes are common and when they will appear and which age-groups are susceptible to produce dysplasia under HPV infection. It will show the distribution of these HPV-subtypes in the different age groups. Lastly, it will give us indirectly impression about the importance of detecting dysplastic cellular changes with conventional cervical cytology and the importance of morphological diagnosis.

## Material and methods

**Figure 1:**
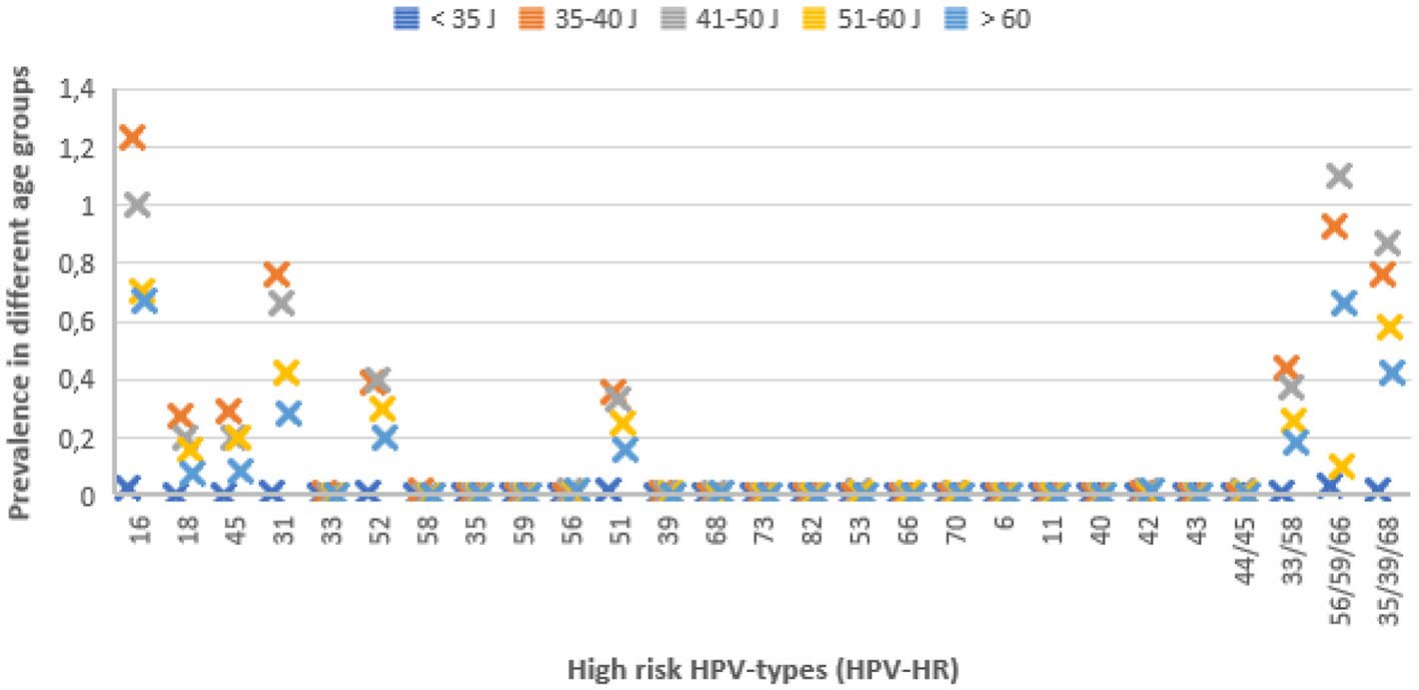
The prevalence of detecting HPV-high risk subtypes in different age groups.

In the Institute for Pathology and Cytology-Schuettorf-Leer-Germany 210.510 samples of women in the screening program were investigated between the beginning of January 2020 until the beginning of January 2021. 63.710 cases under 35 years old, 22.136 cases in the age group 35–40 years old, 34.667 cases in the age group 41–50 years old, 41.276 cases in the age group 51–60 years old and 48.721 cases in the age group older than 60 years old were investigated. The samples were processed for both conventional cytological techniques and for molecular detection and subtyping of HPV-HR according to the advice and measurements of BD-manufacture. We have divided the cases in this study in 5 groups (under 35 years old, 35–40 years old, 41–50 years old, 51–60 years old and above 60 years old). According to the BD manufacture, there were certain HPV-subtypes of high-risk groups studied. These are HPV 16, 18, 45, 31, 33, 52, 58, 35, 59, 56, 51, 39, 68, 73, 82, 53, 66, 70, 6, 40, 42, 43, 44/45, 33/58, 56/59/66, 35/39/68 (Table 1, Fig. 1). In this study, we have studied the histopathological results (Table 2) after colposcopy according to the age subgroups. The histopathological results were subdivided into no dysplasia, cervical intraepithelial neoplasia I (CIN I), cervical intraepithelial neoplasia II (CIN II), cervical intraepithelial neoplasia III (CIN III), squamous cell carcinoma (Sq.c.c), adenocarcinoma in situ (AIS), endometrial carcinoma, endocervical adenocarcinoma and cases without biopsy during the colposcopy (COB). We have used the muenchener classification III (Table 3) as a subgrading system for the cytological specimens. Statistics were calculated with GraphPad Prism, one-way Anova with Kruskal–Wallis test (Prism 5-2007). Significant results would be considered if the *p* value is < 0.05. The approval was granted by the ethics committee (Ethics Committee of the medical association-Hannover-Germany). The samples were anonymous with respect to measurements of data protection. All methods were performed in accordance with the relevant guidelines and regulations.

**Table 1:** The histopathological results distributed in the different age-groups.

Histopath./Age -group	< 35 Y	35–40 Y	41–50 Y (%)	51–60 Y (%)	Over 60 Y (%)
Without dysplasia	0.19%	0.5%	0.56	0.51	0.26
CIN I	0.13%	0.35%	0.36	0.25	0.098
CIN II	0.23%	0.42%	0.26	0.22	0.078
CIN III	0.31%	0.5%	0.33	0.13	0.076
Sq.c.c	0.005%	0	0.009	0.021	0.023
AIS	0.002%	0.005%	0.01	0.005	0.002
Endocervical adeno.ca	0.002%	0.005%	0.014	0.007	0.004
Endometrium ca	0	0	0.003	0.034	0.07
Clinically without biopsy (COB)	0.044%	0.11%	0.12	0.13	0.08

**Table 2:** The most common HPV-subtypes distributed in the different age-groups.

HPV/Age-group	< 35 Y (%)	35–40 Y (%)	41–50 Y (%)	51–60 Y (%)	> 60 Y (%)
56/59/66	0.037	0.93	1.1	0.99	0.66
16	0.03	1.23	1	0.7	0.67
35/39/69	0.017	0.76	0.87	0.58	0.42
51	0.017	0.36	0.33	0.25	0.16
31	0.01	0.76	0.66	0.42	0.28
52	0.007	0.397	0.4	0.3	0.2

**Table 3:** Muenchener classification III with correlation to Bethesda classification.

Group	Definition	Correlation to Bethesda classification
0	Not enough material	Unsatisfactory for evaluation
I	No abnormality	NILM
II-a	No abnormality with a history of abnormal cytological diagnosis	NILM
II-p	Squamous cells with morphological changes but not CIN I	ASC-US
II-g	Endocervical glands with morphological changes e.g. irritation	AGC endocervical NOS
II-e	Endometrium glands in women > 40 years old in 2nd phase of cycle	Endometrial cells
III-p	Atypical squamous cells: CIN II/III or Sq.c.ca are to be considered	ASC-H
III-g	Atypical glandular cells: AIS/Adenocarcinoma are to be considered	AGC endocervical favor neoplastic
III-e	Abnormal endometrial glands especially postmenopausal	AGC endometrial
III-x	Anormal glands not endocervical or endometrial	AGC favor neoplastic
IIID1	Squamous cells with mild dysplasia (CIN I)	LSIL
IIID2	Squamous cells with moderate dysplasia (CIN II)	HSIL
IVa-p	Squamous cells with high dysplasia (CIN III)	HSIL
IVa-g	Endocervical cells with high dysplasia (AIS)	AIS
IVb-p	Squamous cells with high dysplasia and may be invasion	HSIL with features of invasion
IVb-g	Endocervical cells with high dysplasia (AIS) and may be invasion	AIS with features of invasion
V-p	Squamous cell carcinoma	Squamous cell carcinoma (Sq.c.ca)
V-g	Endocervical adenocarcinoma	Endocervical adenocarcinoma
V-e	Endometrial adenocarcinoma	Endometrial adenocarcinoma
V-x	Other type of malignancy	Other malignant neoplasms

*NILM* Negative for intraepithelial lesion or neoplasm;

### Ethical approval

The approval was granted by the ethics committee (Ethics Committee of the medical association-Hannover-Germany). The samples were anonymous with respect to measurements of data protection. All methods were performed in accordance with the relevant guidelines and regulations. We are confirming that informed consent was obtained from all subjects and/or their legal guardian(s).

## Results

### Distribution of HPV-HR-subtypes in relation to the histopathological diagnosis by different studied age groups

Under the age of 35 years (Fig. 1), there were 0.037% of the cases with HPV 56/59/66, followed by 0.03% with HPV16 and then 35/39/69, 51 with frequency of 0.017%. In this age group, there were 0.31% with CIN III, 0.23% with CIN II, 0.19% without dysplasia and 0.13% with CIN I, then (0.005% (Sq.c.c),0.002% for both AIS and endocervical adenocarcinoma, 0.044% (COB). There was no colposcopy and no biopsy in 98.5%. (Table 1).

In the age-group 35–40 years old (Fig. 1), there were HPV16 in 1.23%, then HPV56/59/66 in 0.93% followed by HPV35/39/68 and HPV31 with incidence of 0.76%. In this age-group, there were 0.5% with CIN III or without dysplasia, then 0.42 with CIN II and 0.35% with CIN I. After that, there were 0.11% (COB), 0.005% (AIS and endometrial carcinoma). There were 97.1% of the cases without biopsy and histopathology, about 0.5% without dysplasia after the histopathological investigation (Tables 1 and 2).

In the age-group 41–50 years old (Fig. 1), there were HPV 56/59/66 in 1.1%, HPV 16 in 1%, HPV 35/39/68 in 0.87% and HPV 31 in 0.66%. In this age-group, there were 0.36% with CIN I, 0.33% with CIN III, 0.26% with CIN II, and then 0.014% with endocervical adenocarcinoma, 0.01% (AIS), 0.009% (Sq.c.c) and 0.003% endometrial adenocarcinoma. There were 0.12% with COB and 97.5% without biopsy and histopathology, about 0.56% without dysplasia (Tables 1 and 2).

In the age-group 51–60 years old (Fig. 1), there were HPV 56/59/66 in 0.99%, HPV 16 in 0.7%, HPV 35/39/68 in 0.58%, HPV 31 in 0.42% and HPV 52 in 0.3%. In this age-group, there were 0.25% with CIN I, 0.22% with CIN II, 0.13% with CIN III, then 0.034% with endometrial carcinoma, 0.021% with Sq.c.c, 0.007% with endocervical adenocarcinoma, 0.005% with AIS, 0.13% with COB. About 0.51% without dysplasia and 98% without biopsy and histopathological results (Tables 1 and 2).

In the age-group over 60 years (Fig. 1), there were HPV 16 in 0.67%, HPV 56/59/66 in 0.66%, HPV 35/39/68 in 0.42% and HPV31 in 0.28%. In the histopathological results after colposcopy, there were 0.098% with CIN I, 0,078% with CIN II, 0.076% with CIN III. 0.26% are without dysplasia. 0.023% with squamous cell carcinoma, 0.004% with endocervical carcinoma, 0.07% with endometrial carcinoma and 0.08% clinically without biopsy (Tables 1 and 2).

### Distribution of HPV-HR-subtypes in relation to cytological groups and histopathological diagnosis (Table 4)

**Table 4:** HPV high-risk subtype in relation to cytological group and histopathological diagnosis.

HPV-HR-Subtype/Group-age/histopathological results	Without HB	CIN I	CIN II	CIN III	AIS	Sq.c.ca	Endo.ca
**Under the age of 35 years old**							
HPV 70 (1 = IIg)	1						
HPV-HR-negative (1 = IIIg)					1		
HPV-HR-negative (1 = IVb-g)						1	
HPV-HR-negative (1 = Vg)							1
**35–40 years old**							
HPV18(2),45(2),56/59/66(1),35/39/68(1) = IIg	1	1(18)	1(45)				
HPV 68 (1),56/59/66 (1),45(1) = IIIg				1 (16)	1(18)		
HPV52(1), 18(2), 16(1) = IVa-g			1(−ve)	1(−ve)			
HPV-HR-negative = IVa-g							
**41–50 years old**							
HPV 56/59/66 (1) = IIg	1						
HPV 18 (2), 52(2), 31(1) = IIIg			1(52)	1(52)			1(18)
HPV-HR-negative = IIIg					1(−ve)		
HPV 16(2),18(3),52(1), 33/58(2), 31(1),35/39/68(1) = IVa-g				1(18),1(52),1(33/58),1(35/39/68)	1(16),1(31)	1(16)	1(16)
HPV-HR-negative (1 = Vg)							1(−ve)
**51–60 years old**							
HPV 16(2), 52(1), 35/39/68(1), 56/59/66(2) = IIg	1		1				
HPV 45(1), 33/58(1) = IIIg						1(45)	
HPV-HR-negative = IIIg		2 (−ve)	1 (−ve)				
HPV 18(4), 16(1), 52(1) = IVa-g			1(18)		16(1),18(1)		
HPV18 (2), 16 (1) = IVb-g							1(18)
More than 60 years old							
HPV 16 (1) = IIg	1						
HPV-HR-negative = IIIg	1						
HPV 33/58 (2) = IVa-g		1		1			
HPV 18(1), 52(1), 56/59/66(1) = Vg						1	1

*HB* Histopathological biopsy; *AIS* Adenocarcinoma in situ; *Sq.c.ca* Squamous cell carcinoma; *End. Ca.* Endometrial carcinoma.

Under the age of 35 years, there were 3 cases with negativity for HPV analysis for high-risk subtypes but they were cytologically IIIg, IVb-g, and Vg and histopathologically were AIS, Sq.c.ca and endometrial carcinoma respectively (Table 4).

Between 35 and 40 years old: One case was negative for HPV analysis for high-risk-subtypes but was cytologically IIIg and histopathologically CIN II. Two cases were with HPV (18) but cytologically were IIg and IIIg then histopathologically CIN I and AIS. One case was with HPV (45) but cytologically IIg and histopathologically CIN II.

Between 41 and 50 years old: One case was negative in HPV-HR-analysis but cytologically was IIIg and histopathologically AIS. A case was with HPV (52) and cytologically was IIIg and histopathologically CIN II. 4 cases were with HPV (16) and cytologically were IVa-g but histopathologically were AIS (2 cases), endocervical adenocarcinoma (1 case) and Sq.c.ca (1 case).

Between 51and 60 years old: Three cases were negative in HPV-HR-analysis but cytologically were IIIg and histopathologically were CIN I and CIN II. Three cases were with HPV (18) and cytologically were IVa-g and IVb-g but histopathologically either CIN II or AIS or endometrial carcinoma.

More than 60 years old: Two cases were with HPV 33/35 and cytologically were IVa-g but histopathologically was CIN I and CIN III.

## Discussion

Cervical cancer represents 570,000 new cases per year and accounts for the vast majority of all HPV-attributable cancer cases worldwide. 311,000 women died of the disease^4^. WHO had announced shocking deductions: one woman died of cervical cancer every 2 min. worldwide (WHO^1^). Currently studies point out that 20–25% of all human malignancies are related to micro-organism infections. Among these cancer-related pathogens, the human papillomavirus (HPV) has a prominent position, since the virus is responsible for about 30% of all infectious agent-related cancers as stated by Araldi RP and colleagues, 2018^5^. Another work of de Martel C. and Colleagues, 2017^6^, who have documented that 4.5% of all cancers worldwide (630,000 (4.5%)) new cancer cases per year worldwide are attributable to HPV. The association of infection with HPV16/18 and HPV 6/11/16/18/31/33/45/52/58 was also estimated. Other organs like vagina or anus or penis in men show also HPV-associated lesions. The most common types worldwide are HPV 16 and 18, which are the main types linked to carcinogenesis as reported by Palefsky JM, 2017^7^, while HPV types 31, 33, 35, 39, 45, 51, 52, 56, 58, 59, 66, 68, 73, and 82 are also oncogenic and responsible for little number of cases as reported by Schiffman and colleagues, 2009^8^. It is well documented that nearly 90% of incident HPV infections are not detectable within a period of 2 years from the acquisition of infection and persist only in a small proportion as reported by Bosch and colleagues 2002^9^. This is somehow compared with our study but not completely agree with the work of Palefsky JM, 2016^7^, we have noticed among 210.510 cases about 1.1% with HPV-subtypes 56/59/66 in the age group 41–50 years old. These subtypes were detected with a rate of 0.93% in the age group 35–40 years old and with a rate of 0.99% in the age group 51–60 years old, which means that the peak of incidence was in the middle age group. This might explain the chronicity of this infection with these subtypes, that may lead to cervical dysplasia, as we have detected later in the histopathology results, that the peak of occurrence of CIN I (0.36%) was in the age group 41–50 years as well as AIS (0.01%) and endocervical adenocarcinoma of (0.014%). HPV-subtype 16 has an incidence in our work of 1.32% in the age-group 35–40 years and a little bit lower (1%) in the age group 41-50Ys, 0.7% in the age-group 51–60 years and 0.67% in the age-group of more than 60 years, which may explain the highest incidence of CIN I (0.35%), CIN II (0.42%) and CIN III (0.5%) in the age-group 35–40 years. The incidence of these lesions was small in the higher age groups. It should be noted that in the age-group 35–40 years, there was no dysplasia in 0.5%. This may explain that not every woman with HPV infection should develop dysplasia and this confirms the statement of Schiffmann and colleagues, 2018^10^. They have also concluded that the true value of HPV primary screening is uncertain since a positive HPV test reflecting a developed cervical carcinoma may not lead to disease prevention. More confounding is the likelihood of HPV-negative, cytology-positive cervical cases, thus, the diagnostic value embedded in co-testing Pap-test models. In our work, there is no significance between occurrence of cervical dysplasia and without dysplasia despite of detection of HPV-HR subtypes (*p* value = 0.1754). From Australia’s screening program with reflex cytology, there were high-grade lesions in 24.3% confirmed, which underscores the benefits through cell-morphology-based co-testing, and the potential deficits in HPV DNA screening. Related studies emphasize the need to minimize the false-positives arising from HPV DNA screening to avert harms to women, such as clinical overreaction, overtreatment and psychological distress, which are all associated with profound yet avertable social costs as stated by Schiffmann and colleagues, 2019^11^. We showed in our study that the incidence of HPV-subtypes was low and matched with the occurrence of squamous cell carcinoma or endocervical adenocarcinoma. HPV 52, HPV 16, HPV 58, HPV 51, and HPV 39 were the most common genotypes accounting for 22.8%, 22.3%, 20.0%, 14.3%, and 13.6% of cases, respectively. The highest infection rates were found in 20–30-year-old patients (35.1%). HPV 16 infection was the highest in the age-group 31–40 years, and HPV 52, HPV 58, and HPV 39 infections were highest in the age-group 20–30 years in the work of Shuang Lu and colleagues, 2015^12^. The results showed that HPV 52 and HPV 16 were the most common subtypes found in 22.8% and 22.3% respectively of the study participants. HPV 58, HPV 51, and HPV 39 were also common and occurred in 20%, 14.3% and 13.6% of patients, respectively as reported by Shuang Lu and colleagues 2015^12^. These results should be evaluated carefully as the incidence of HPV 52 was too small in all age groups with maximum of 0.4% in the age-group 41–50 years. The variability between nations may lead to variation in the results. The most common found subtypes were HPV 16 and HPV 18. It is found that people in Europe or South/Central America were more likely to be infected with HPV 18 than those in Asia. Other common subtypes were HPV 31 in Europe, HPV 33 in South/Central America, and several HPV genotypes (HPV 39, HPV 51, HPV 53, HPV 56, HPV 59, and HPV 66) were in North America compared with other regions as stated by Bosch and colleagues, 1995^13^. These results should be evaluated carefully, as HPV-18 was totally irrelevant in the age groups and has no association with the development of cervical carcinoma. Shuang Lu and colleagues 2015^12^ have stated that the highest infection rates were found in patients 20–30 years old. At 31–40 years of age, the infection rates of HPV 16 were significantly higher than HPV 52 and HPV 58 infection rates. These differences may result from different clearance rates of HPV 52, HPV 58, and HPV 16. Low grade (I) cervical lesions, in the work of Shuang Lu and colleagues 2015^12^, were most common in women 31–40 years old, and the prevalence of degree II + cervical lesions were highest in women 41–50 years old. In our study, we have detected the highest infection with HPV subtype 16 in age group 35–40 years old, followed by HPV 56/59/66 in age group 41–50 years old. We have also found that CIN I was high in the age group 41–50 years old (0.36%). CIN II and CIN III were highest in the age groups 35–40 and 41–50 years old. Under the age of 35 years old, we have also detected the same rates of CIN I, CIN II and CIN III of 0.13%, 0.23% and 0.31% respectively. Poljak^14^ has stated that HPV-based cervical cancer screening is more sensitive than cytology for detecting underlying CIN2+, CIN3 + and cervical cancer, is more accurate and objective, is less variable than cytology, requires less training, shows better reproducibility, offers a possibility of self-sampling for non-attenders, and provides safe extension of screening intervals in women with a negative screening result. Another work of Maver and Poljak^15^, that has proposed for HPV-based screening in some European countries and Australia. The work of Miller RA and colleagues^16^ has documented HPV-negative cervical cancers, which would be not detected, if we depend only on primary screening with HPV-test. Based on our work and on the work of Schiffmann and his colleagues, 2018^11^ as well as the results of HPV-screening, we come to the conclusion that the only use of HPV-subtyping is not a secure method and a protective way for women. In the work of Baay M.F.D and his colleagues^17^^,^ there were 13% HPV-negative cancers. There are too many HPV-positive cases, which have been psychologically impaired with higher costs, although they have no cervical epithelial changes during the HPV-infection. There are too many HPV-negative cases, which develop cervical cancer. We have the opinion and are convinced that the screening should be both morphologically via cytological examination and may be with adding immunocytochemistry as presented by Abbas and his colleagues, 2022^2^ and the HPV-Genotyping as an automated fast method, but the results of both should be respectively analyzed and both clinicians and patients should be accordingly advised.

## Data Availability

The datasets used and/or analysed during the current study available from the corresponding author on reasonable request.
